# Supplementary material for: acCRISPR: an activity-correction method for improving the accuracy of CRISPR screens
Source: Commun Biol. 2023 Jun 8;6:617. doi: 10.1038/s42003-023-04996-8 (PMC10250353; doi:10.1038/s42003-023-04996-8)
Supplement: Supplementary file 2 — Supplementary Information [file 42003_2023_4996_MOESM2_ESM.pdf]

Supplementary Information

acCRISPR: An activity-correction method for improving the accuracy of CRISPR screens

Adithya Ramesh[1,#], Varun Trivedi[1,#], Sangcheon Lee[1,#], Aida Tafrishi[1], Cory Schwartz[1,†], Amirsadra Mohseni[2], Mengwan Li[1], Stefano Lonardi[2,3], and Ian Wheeldon[1,3,4*]

1. Department of Chemical and Environmental Engineering, University of California, Riverside, CA 92521, USA
2. Department of Computer Science, University of California, Riverside, CA 92521, USA
3. Integrative Institute for Genome Biology, University of California, Riverside, CA 92521, USA
4. Center for Industrial Biotechnology, University of California, Riverside, CA 92521, USA

# These authors contributed equally
† Current address: iBio Inc., San Diego, CA
* Corresponding author: wheeldon@ucr.edu

Supplementary Figure 1: acCRISPR analysis of Cas12a growth screens in *Yarrowia lipolytica.*

Supplementary Figure 2: Experimental validation of CRISPR-Cas9 and CRISPR-Cas12a essential and non-essential genes from acCRISPR analysis.

Supplementary Figure 3: Essential gene comparison to *S. cerevisiae* and *S. pombe.*

Supplementary Figure 4: Performance of acCRISPR on the Cas12a screening dataset with predicted sgRNA activities.

Supplementary Figure 5: acCRISPR corrected Tolerance Scores (TS) for salt tolerance screens.

Supplementary Figure 6: Number of significant genes at different levels of activity correction for high salt tolerance screens.

Supplementary Figure 7: Experimental validation of high salt tolerance genes from acCRISPR analysis.

Supplementary Figure 8: CRISPR-Cas9 and -Cas12a FS distributions on days 2, 4 and 6.

Supplementary Figure 9: Schematic and sequence information of Cas9 and Cas12a amplicons for NGS.

Supplementary Table 1: CS threshold data for Cas9 and Cas12a screens.

Supplementary Table 2: Yeast strains used in this study.

Supplementary Table 3: Plasmids used for genome wide CRISPR screens.

Supplementary Table 4: Sequences of primers used in this study.

Supplementary Table 5: Transformation efficiencies measured as x10^6 transformants, for all replicates in the control and treatment strains.

Supplementary Table 6: Primers used for NGS fragment amplification (Cas12a).

Supplementary Table 7: Primers used for NGS fragment amplification (Cas9).

Supplementary Table 8: Parameters for bioinformatics tools used in the analysis of NGS reads (Cas12a).

Supplementary Table 9: Parameters for bioinformatics tools used in the analysis of NGS reads (Cas9).

Supplementary Table 10: List of sgRNA (& associated cutting scores) used for validation of essential genes, non-essential genes, and significant genes for salt tolerance.

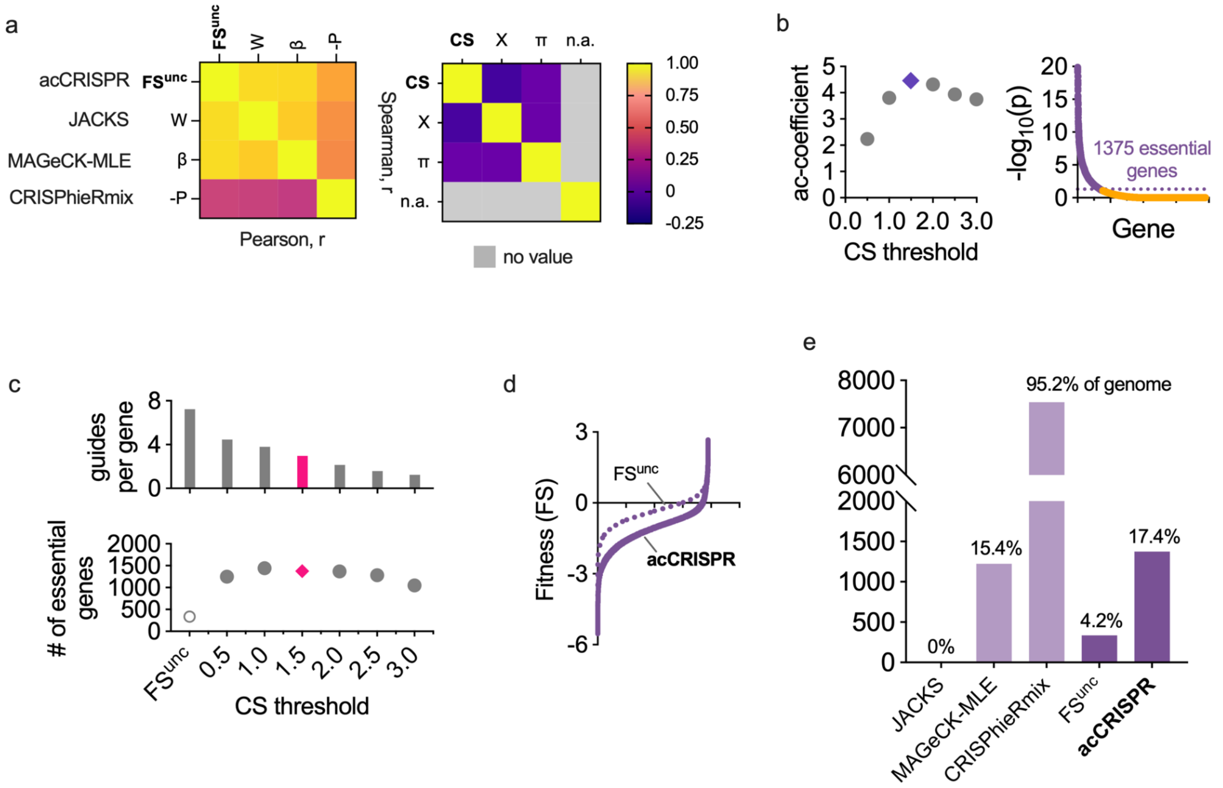

Supplementary Figure 1. acCRISPR analysis of Cas12a growth screens in *Yarrowia lipolytica*. (**a**) Heat-maps showing Pearson (below diagonal) and Spearman (above diagonal) coefficients of fitness effects (uncorrected FS (FS^{unc}), W, β & -P; left) and sgRNA cutting efficiencies (CS, X and π; right) and from acCRISPR and three established essential gene identification algorithms, JACKS, MAGeCK-MLE and CRISPhieRmix. **(b)** ac-coefficient is calculated with increasing CS threshold values and maximum value is represented by the purple datapoint. Genes with a p-value < 0.05 were classified as essential at the maximum ac-coefficient value. (**c**) Average number of sgRNA per gene and the number of essential genes predicted with increasing CS threshold. The number of essential genes predicted for the corrected and uncorrected analyses. The data points colored in pink are the guides per gene and number of essential genes determined at the optimum CS threshold. (**d**) Fitness scores of genes with (solid line) and without (dashed line) acCRISPR processing with a CS threshold of 1.5. (**e**) Number of essential genes identified by JACKS [1], MAGeCK-MLE [2], CRISPhieRmix [3], FS^{unc}, and acCRISPR along with the percentage of total genes in the genome are reported.

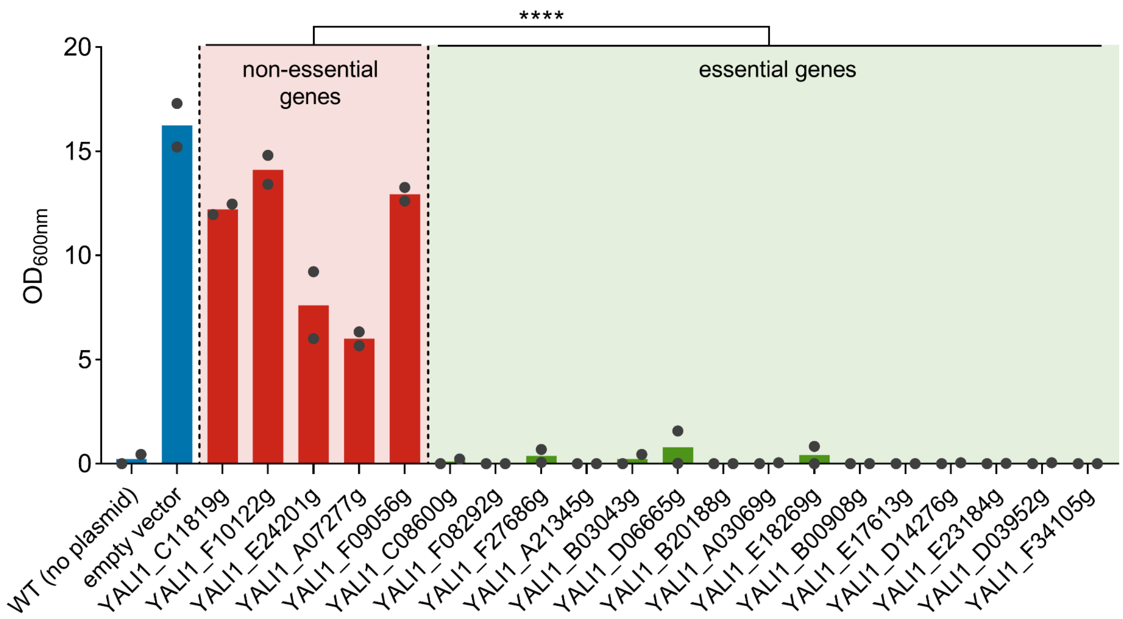

Supplementary Figure 2. Experimental validation of CRISPR-Cas9 and CRISPR-Cas12a essential and non-essential genes from acCRISPR analysis. Final OD of cells containing single knockouts of 5 non-essential genes (red bars) and 15 essential genes (green bars) from the consensus set. Of the 15 selected for validation, 12 were called as essential genes in all 3 screens (Cas9, Cas12a and transposon [4]). The other three genes, YALI1_B03043g, YALI1_E18269g and YALI1_F34105g, were called as essential only in the Cas9 and Cas12a screens. Cells were grown in SD-Leu for 16 hrs post sgRNA transformation, followed by subculturing in fresh media and growth for another 2 days before measuring final OD. An empty vector control (blue bar) was used to show growth in absence of any knockout. The PO1f strain containing no plasmid (indicated as WT; leftmost bar) was used as no growth, negative control. Each bar represents mean of two biological replicates (n = 2), and data points represent OD of each individual replicate in the respective sample (****$p < 0.0001$; one-tailed unpaired t-test).

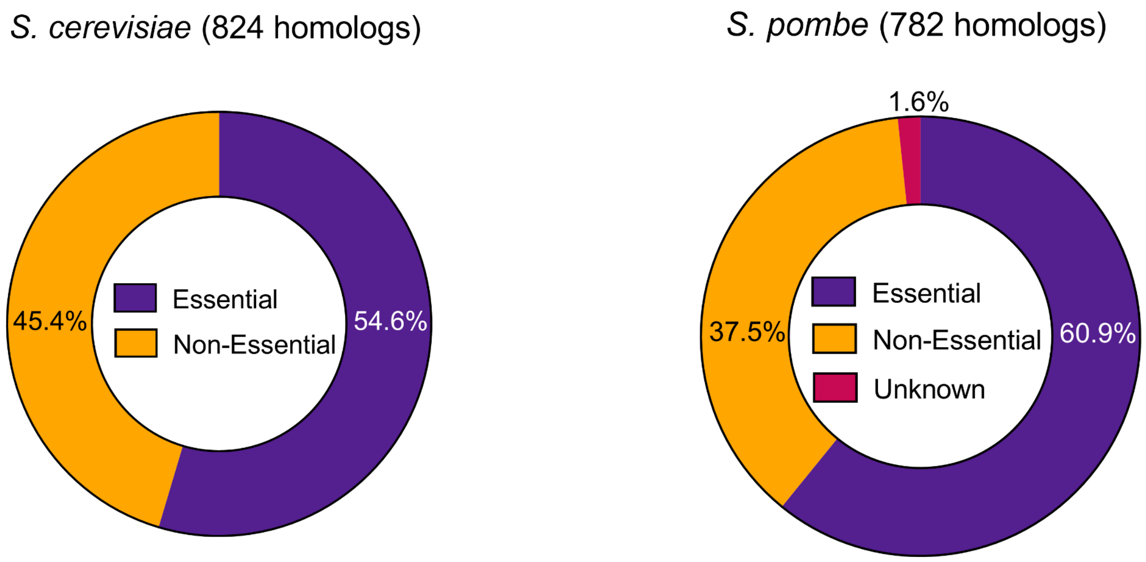

Supplementary Figure 3. Essential gene comparison to *S. cerevisiae* and *S. pombe*. Pie charts indicating the percentage of homologs in the *Y. lipolytica* consensus set that are essential, non-essential and have unknown essentiality in *S. cerevisiae* (824 homologs) and *S. pombe* (782 homologs).

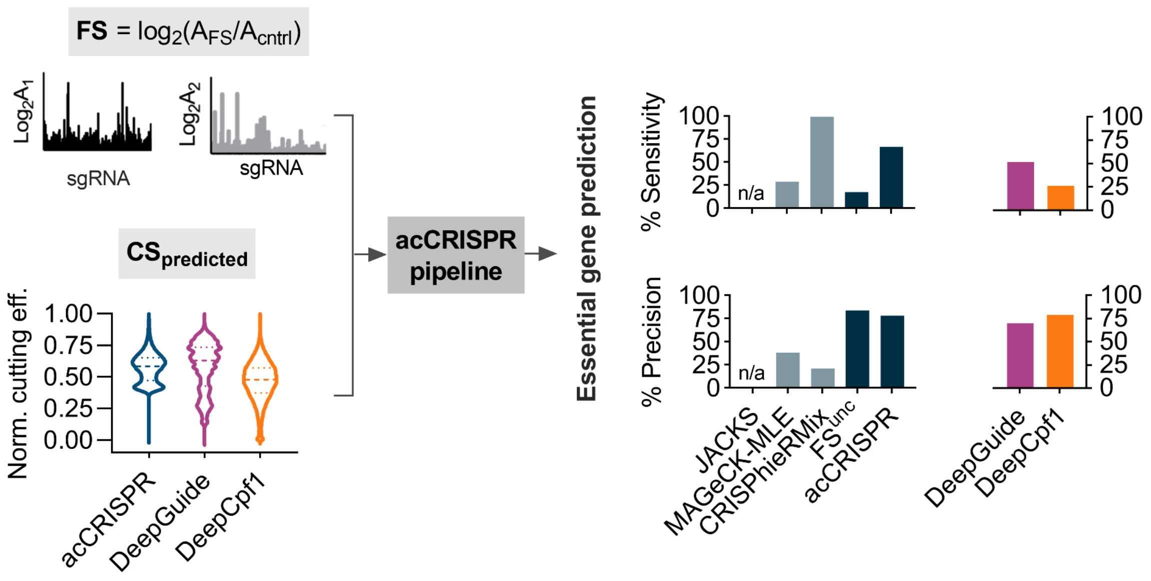

Supplementary Figure 4. Performance of acCRISPR on the Cas12a screening dataset with predicted sgRNA activities. Essential genes were determined with acCRISPR utilizing FS along with predicted sgRNA activities from DeepGuide [5] and DeepCpf1 [6]. The violin plot shows min-max normalized sgRNA activity distributions of experimental CS determined by acCRISPR and those from DeepGuide and DeepCpf1. The % sensitivity and % precision in identifying genes from the consensus set is shown (right). Bars indicate the values of these two metrics for each prediction tool as well as for JACKS [1], MAGeCK-MLE [2], CRISPhieRmix [3], uncorrected FS (FS only) and acCRISPR.

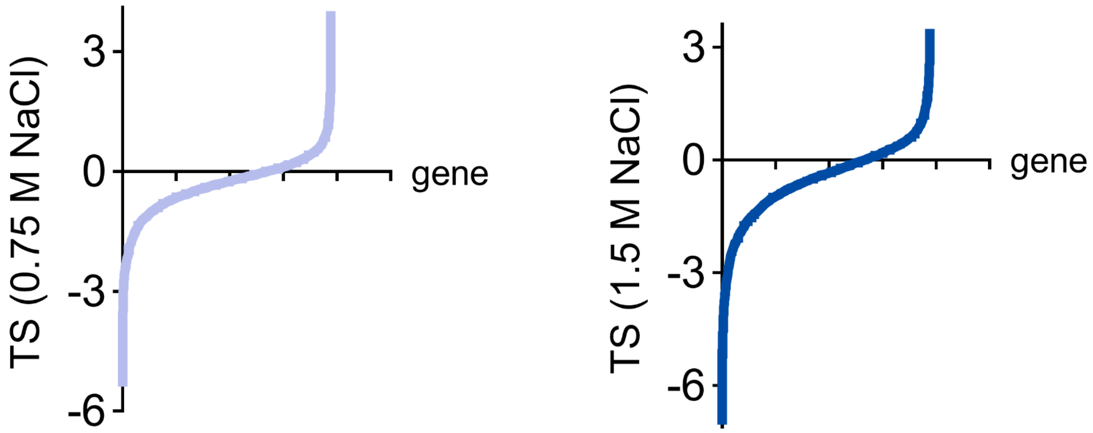

Supplementary Figure 5. acCRISPR corrected Tolerance Scores (TS) for salt tolerance screens. S-curves showing tolerance scores of genes at a CS threshold of 4.5 for the two salt stress conditions – 0.75 M NaCl (left) and 1.5 M NaCl (right).

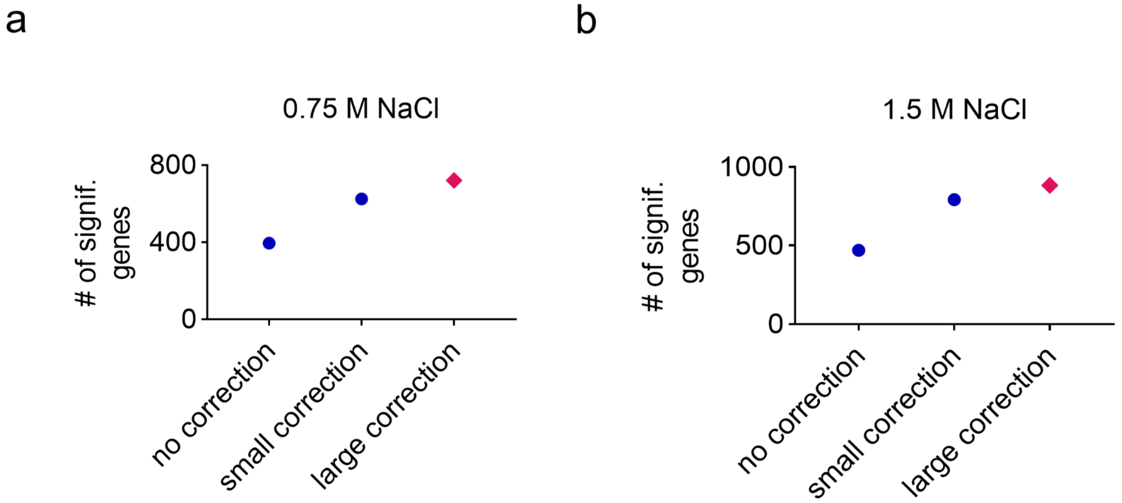

Supplementary Figure 6. Number of significant genes at different levels of activity correction for high salt tolerance screens. Dark blue points represent the number of significant genes predicted by acCRISPR without CS correction and with a small CS correction (CS-threshold = 1.0), while pink diamonds indicate the number of predicted significant genes with a large CS correction (CS-threshold = 4.5, i.e., optimum CS-threshold) for NaCl concentrations of (**a**) 0.75 M, and (**b**) 1.5 M.

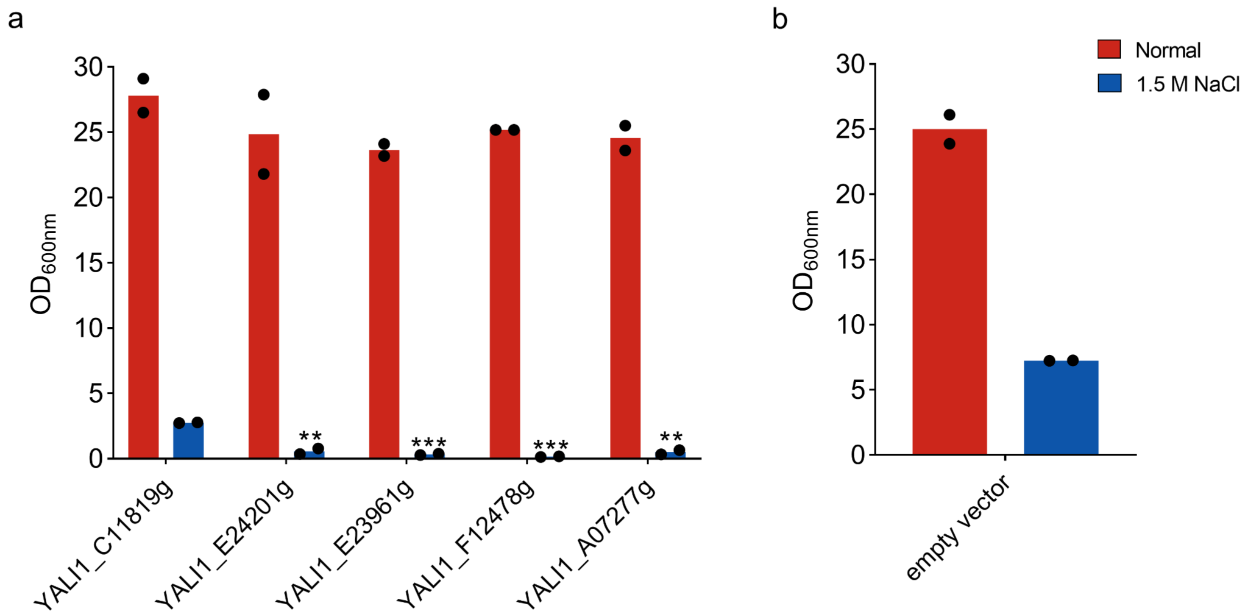

Supplementary Figure 7. Experimental validation of high salt tolerance genes from acCRISPR analysis. Bars represent final OD of (**a**) 5 single gene knockouts (*i.e.*, 4 significant genes and a non-significant gene, YALI1_C11819g), and (**b**) an empty vector control, grown in absence (normal; red bars) and presence (1.5 M NaCl; blue bars) of high salt conditions. Cells were grown in SD-Leu for 16 hrs post sgRNA transformation, followed by subculturing in fresh media (containing 1.5 M NaCl for the high salt condition) and growth for 4 days before measuring final OD. Bars indicate mean of two biological replicates (n = 2), and data points represent OD of each individual replicate in the respective sample (**p < 0.01, ***p < 0.001 ; one-tailed unpaired t-test).

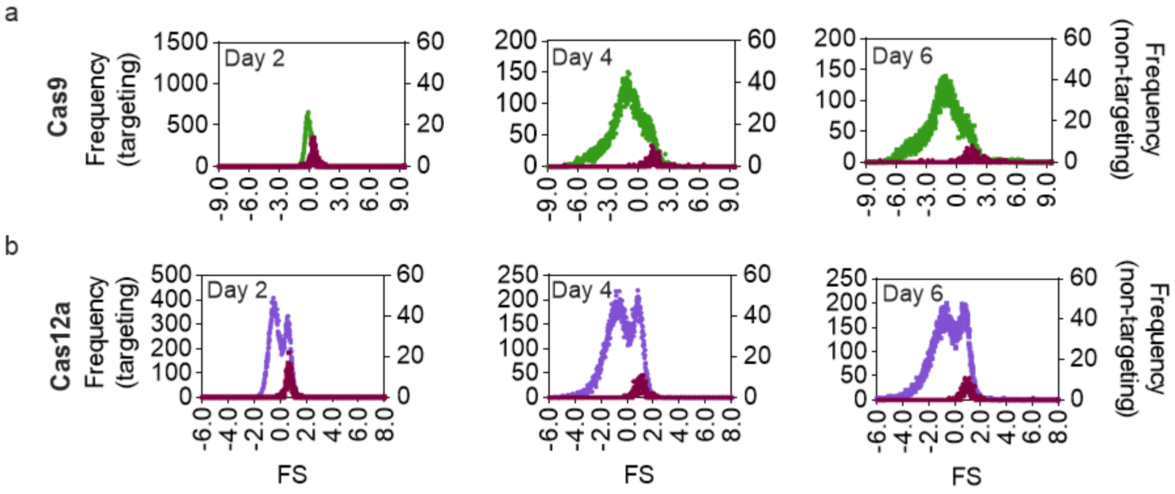

Supplementary Figure 8. CRISPR-Cas9 and -Cas12a FS distributions on days 2, 4 and 6. Green and purple distributions plotted on the left y-axis show FS of all targeting sgRNA in the library, while the dark red distributions plotted on the right y-axis represents the non-targeting populations. (a) Histogram of sgRNA FS values in the Cas9 dataset. (b) Histogram of sgRNA FS values in Cas12a dataset.

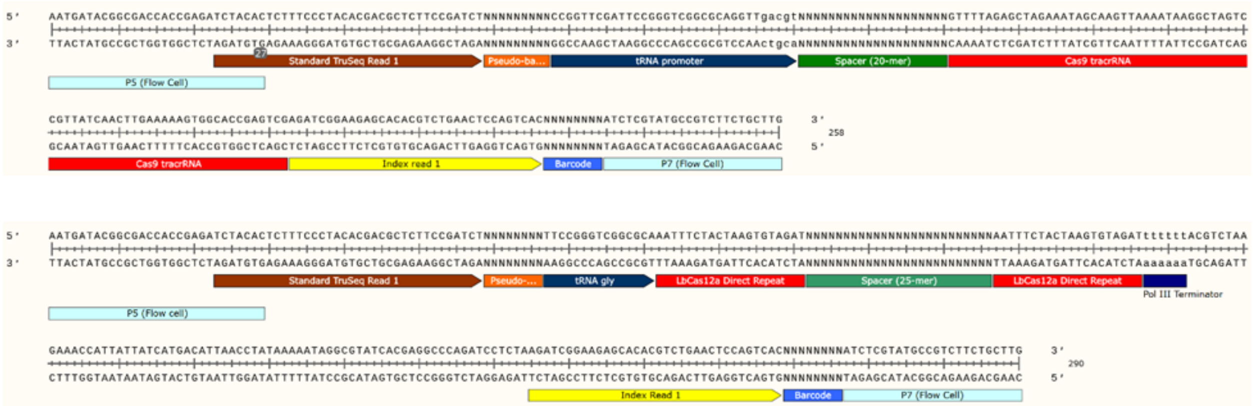

Supplementary Figure 9. Schematic and sequence information of Cas9 (top) and Cas12a (bottom) amplicons for NGS. Amplicons contain: (i) P5 and P7 sequences (light blue) that are necessary for binding with the flow cell in Illumina sequencers, (ii) TruSeq adapter (brown) for binding of the sequencing primer, (iii) a portion of $tRNA^{gly}$ (black) expressing the sgRNA, (iv) Cas9 or Cas12 spacer (green) (v) Cas12a associated direct repeats or a portion of the Cas9 tracrRNA sequence (red), (vi) Universal 8 bp Illumina barcodes (blue), (vii) Index read 1 sequence for the binding of primers to sequence the Illumina barcodes, and (viii) 4-9 nt pseudo-barcodes (orange) at the 5' end between the TruSeq and $tRNA^{gly}$ which help demultiplex replicates that contain the same Illumina barcode.

Supplementary Table 1. CS threshold data for Cas9 and Cas12a screens. The CS threshold values used to generate 'CS-corrected' libraries and the optimum cutoff value for Cas9 and Cas12a datasets.

**Cas9 Screen**	**Value**			
**Cutting efficiency score**	**Lowest cutoff**	**Highest cutoff**	**Step size**	**Optimum cutoff**
Experimental CS	0.5	6.0	0.5	4.5
DeepGuide CS	0.5	6.0	0.5	4.0
Designer v1	0.108	0.892	0.098	0.402
Designer v2	20.209	78.441	7.279	49.325
CRISPRspec	1.215	39.175	4.745	15.45
CRISPRscan	0.491	0.739	0.031	0.553
SSC	0.301	0.789	0.061	0.484
uCRISPR	10.045	90.005	9.995	70.015

**Cas12a Screen**	**Value**			
**Cutting efficiency score**	**Lowest cutoff**	**Highest cutoff**	**Step size**	**Optimum cutoff**
Experimental CS	0.5	3.0	0.5	1.5
DeepGuide CS	0.5	2.5	0.5	1.0
DeepCpf1	10	90	10	40

Supplementary Table 2. Yeast strains used in this study.

**Yeast strain genotype**	**Phenotype**
PO1f (MatA, *leu2-270*, *ura3-302*, *xpr2-322*, *axp-2*)	Wild type strain
PO1f *Δku70*	PO1f with disrupted KU70, which facilitates the non-homologous end joining DNA repair pathway
PO1f UAS1B8-TEF(136)-Cas9 -CycT::A08	PO1f expressing *Y. lipolytica* codon optimized Cas9 gene at the A08 locus
PO1f UAS1B8-TEF(136)-LbCas12a -CycT::A08	PO1f expressing *Y. lipolytica* codon optimized LbCas12a gene at the A08 locus
PO1f *Δku70* UAS1B8-TEF(136)-Cas9 -CycT::A08	*KU70* disrupted in Cas9 integrated PO1f strain
PO1f *Δku70* UAS1B8-TEF(136)-LbCas12a -CycT::A08	*KU70* disrupted in LbCas12a integrated PO1f strain

Supplementary Table 3. Plasmids used for genome wide CRISPR screens.

Plasmid name	Reference	Function
pCpf1_yl	[7]	Plasmid for CRISPR-LbCas12a based gene editing in *Y. lipolytica*
pCRISPRyl (Addgene #70007)	[8]	Plasmid for CRISPR-Cas9 based gene editing in *Y. lipolytica*
pLbCas12ayl	This study and [5]	Plasmid for CRISPR-LbCas12a based gene editing in *Y. lipolytic*a. sgRNA is flanked on either end by the direct repeat, to allow sgRNAs to end in T residues without being construed as part of the PolyT terminator
pHR_A08_hrGFP (Addgene #84615)	[9]	Plasmid containing homology arms for integration of hrGFP into the A08 locus
pHR_A08_LbCas12a	This study and [5]	Plasmid containing homology arms for integration of LbCas12a into the A08 locus
pHR_A08_Cas9	[10]	Plasmid containing homology arms for integration of Cas9 into the A08 locus
pLbCas12ayl-GW	This study and [5]	Vector containing sgRNA expression cassette for cloning Cas12a sgRNA library. (Does not contain Cas12a expression cassette)
pCas9yl-GW	[10]	Vector containing sgRNA expression cassette for cloning Cas9 sgRNA library. (Does not contain Cas9 expression cassette)
pCRISPRyl_KU70	This study and [11]	CRISPR plasmid for the disruption of KU70

Supplementary Table 4. Sequences of primers used in this study.

Primer name	Primer Sequence
ExtraDR-F	CGGCGCAAATTTCTACTAAGTGTAGACTAGTAATTTCTACTAAGTGTAGATTTTTTACGTCTAAGAAACCATTATT
ExtraDR-R	AATAATGGTTTCTTAGACGTAAAAAATCTACACTTAGTAGAAATTACTAGTCTACACTTAGTAGAAATTTGCGCCG
Cpf1-Int-F	TGCCTGGAGCCGAGTACGGCATTGATTACTAGTCCGGGTTCGAAGGTACCAAG
Cpf1-Int-R	TTAGGCTGGGTCTCGAGAGCAAAGAAGCCTAGGGCAAATTAAAGCCTTCGAGCG
BRIDGE-F	CTAAATTTGATGAAAGGGGGATCCCCCGGGTGGCGTAATCATGGTCATAGCTGTTTCCTG
BRIDGE-R	CAGGAAACAGCTATGACCATGATTACGCCACCCGGGGGATCCCCCTTTCATCAAATTTAG
A08-Seq-F	AGCCGAGTACGGCATTGAT
A08-Seq-R	TCAATGTAGCCTCCTCCAACC
Tef_Seq-F	GTTGGGACTTTAGCCAAG
Lb1-R	CTTCTGCTTGGTCTTCTGGTTG
Lb2-F	AACCTGTACAACCAGAAGACCAAG
Lb3-F	AAGGAGACCAACCGAGACGAG
Lb4-F	AACCTGCACACCATGTACTTCAAG
Lb5-F	CCAGATCACCAACAAGTTCGAGTC
M13-F	GTAAAACGACGGCCAGT
InversePCR-F	TTTTTTACGTCTAAGAAACCATTATTATCATGACATTAACCT
InversePCR-R	TGCGCCGACCCGGAATCGAACCGGGGGCCC
OLS-F	GTTTAGTGGTAAAATCCATCGTTGCCATCG
OLS-R	GATACGCCTATTTTTATAGGTTAATGTCATG
qPCR-GW-F	TTATGAACTGAAAGTTGATGGC
qPCR-GW-R	TCACACAGGAAACAGCTATG
Cr_1250	TATAAGAATCATTCAAAGGCGCGCATGGATAAGAAATACTCCATTGGCCTG
Cr_1254	ATAACTAATTACATGAGGCTAGCTTACAGCATGTCCAGATCGAAATCG
Sg-Seq	CTTCGACTCTAGAGGATCTGG

Supplementary Table 5. Transformation efficiencies measured as x10^6 transformants, for all replicates in the control and treatment strains.

**Cas9 Screen**	**Replicate Transformation Efficiency (x10^6 transformants)**		
**Strain**	**R1**	**R2**	**R3**
PO1f	12.35	11.39	15.80
PO1f Cas12a	11.42	8.29	10.64
PO1f Cas12a Δku70	6.79	7.33	7.08

**Cas12a Screen**	**Replicate Transformation Efficiency (x10^6 transformants)**		
**Strain**	**R1**	**R2**	**R3**
PO1f Δku70	6.89	6.21	5.43
PO1f Cas12a Δku70	5.06	4.29	4.41
PO1f	11.93	8.28	4.23
PO1f Cas12a	6.32	5.47	6.11

Supplementary Table 6. Primers used for NGS fragment amplification (Cas12a)

Primer name	Primer Sequence	Illumina Barcode (Reverse primer) / Pseudo-Barcode (Forward primer) for demultiplexing
ILU1-F	AATGATACGGCGACCACCGAGATCTACACTCTTTCCCTACACGACGCTCTTCCGATCTTTCCGGGTCGGCGCAAATTTC	^TTCCGG
ILU2-F	AATGATACGGCGACCACCGAGATCTACACTCTTTCCCTACACGACGCTCTTCCGATCTAGATCGGGTCGGCGCAAATTTCT	^AGATCG
ILU3-F	AATGATACGGCGACCACCGAGATCTACACTCTTTCCCTACACGACGCTCTTCCGATCTGCTATTCGGGTCGGCGCAAATTTCT	^GCTATT
ILU4-F	AATGATACGGCGACCACCGAGATCTACACTCTTTCCCTACACGACGCTCTTCCGATCTCAGGACTACGGGTCGGCGCAAATTTCT	^CAGGAC
ILU1-R	CAAGCAGAAGACGGCATACGAGATTCGCCTTGGTGACTGGAGTTCAGACGTGTGCTCTTCCGATCTTAGAGGATCTGGGCCTCGTGATAC	CAAGGCGA
ILU2-R	CAAGCAGAAGACGGCATACGAGATGACGAGAGGTGACTGGAGTTCAGACGTGTGCTCTTCCGATCTTAGAGGATCTGGGCCTCGTGATAC	CTCTCGTC
ILU3-R	CAAGCAGAAGACGGCATACGAGATAGACTTGGGTGACTGGAGTTCAGACGTGTGCTCTTCCGATCTTAGAGGATCTGGGCCTCGTGATAC	CCAAGTCT
ILU4-R	CAAGCAGAAGACGGCATACGAGATCTGTATTAGTGACTGGAGTTCAGACGTGTGCTCTTCCGATCTTAGAGGATCTGGGCCTCGTGATAC	TAATACAG
ILU5-R	CAAGCAGAAGACGGCATACGAGATCCTGAACCGTGACTGGAGTTCAGACGTGTGCTCTTCCGATCTTAGAGGATCTGGGCCTCGTGATAC	GGTTCAGG
ILU6-R	CAAGCAGAAGACGGCATACGAGATATCAGGTTGTGACTGGAGTTCAGACGTGTGCTCTTCCGATCTTAGAGGATCTGGGCCTCGTGATAC	AACCTGAT
ILU7-R	CAAGCAGAAGACGGCATACGAGATTAGGTGACGTGACTGGAGTTCAGACGTGTGCTCTTCCGATCTTAGAGGATCTGGGCCTCGTGATAC	GTCACCTA

ILU8-R	CAAGCAGAAGACGGCATACGAGATCGAACAGTGTGACTGGAGTTCAGACGTGTGCTCTTCCGATCTTAGAGGATCTGGGCCTCGTGATAC	ACTGTTCG
ILU9-R	CAAGCAGAAGACGGCATACGAGATGTTCGATCGTGACTGGAGTTCAGACGTGTGCTCTTCCGATCTTAGAGGATCTGGGCCTCGTGATAC	GATCGAAC
ILU10-R	CAAGCAGAAGACGGCATACGAGATACCTAGCTGTGACTGGAGTTCAGACGTGTGCCTTCCGATCTTAGAGGATCTGGGCCTCGTGATAC	AGCTAGGT
ILU11-R	CAAGCAGAAGACGGCATACGAGATAGAGATGAGTGACTGGAGTTCAGACGTGTGCTCTTCCGATCTTAGAGGATCTGGGCCTCGTGATAC	TCATCTCT
ILU12-R	CAAGCAGAAGACGGCATACGAGATCTGGACTTGTGACTGGAGTTCAGACGTGTGCTCTTCCGATCTTAGAGGATCTGGGCCTCGTGATAC	AAGTCCAG

Supplementary Table 7. Primers used for NGS fragment amplification (Cas9)

Primer name	Primer Sequence	Illumina Barcode (Reverse primer) / Pseudo-Barcode (Forward primer) for demultiplexing
**Cr_1665**	AATGATACGGCGACCACCGAGATCTACACTCTTTCCCTACACGACGCTCTTCCGATCTAGTCCGGTTCGATTCCGGGTC	^AGTCCG
**Cr_1666**	AATGATACGGCGACCACCGAGATCTACACTCTTTCCCTACACGACGCTCTTCCGATCTGTAGTCCGGTTCGATTCCGGGTC	^GTAGTC
**Cr_1667**	AATGATACGGCGACCACCGAGATCTACACTCTTTCCCTACACGACGCTCTTCCGATCTCAGTAGTCCGGTTCGATTCCGGGTC	^CAGTAG
**Cr_1668**	AATGATACGGCGACCACCGAGATCTACACTCTTTCCCTACACGACGCTCTTCCGATCTTCCAGTAGTCCGGTTCGATTCCGGGTC	^TCCAGT
**Cr_1669**	CAAGCAGAAGACGGCATACGAGATTCGCCTTGGTGACTGGAGTTCAGACGTGTGCTCTTCCGATCTCGACTCGGTGCCACTTTTTCAAG	CAAGGCGA
**Cr_1670**	CAAGCAGAAGACGGCATACGAGATATAGCGTCGTGACTGGAGTTCAGACGTGTGCTCTTCCGATCTCGACTCGGTGCCACTTTTTCAAG	GACGCTAT
**Cr_1671**	CAAGCAGAAGACGGCATACGAGATGAAGAAGTGTGACTGGAGTTCAGACGTGTGCTCTTCCGATCTCGACTCGGTGCCACTTTTTCAAG	ACTTCTTC
**Cr_1672**	CAAGCAGAAGACGGCATACGAGATATTCTAGGGTGACTGGAGTTCAGACGTGTGCTCTTCCGATCTCGACTCGGTGCCACTTTTTCAAG	CCTAGAAT
**Cr_1673**	CAAGCAGAAGACGGCATACGAGATCGTTACCAGTGACTGGAGTTCAGACGTGTGCTCTTCCGATCTCGACTCGGTGCCACTTTTTCAAG	TGGTAACG
**Cr_1709**	CAAGCAGAAGACGGCATACGAGATGTCTGATGGTGACTGGAGTTCAGACGTGTGCTCTTCCGATCTCGACTCGGTGCCACTTTTTCAAG	CATCAGAC
**Cr_1710**	CAAGCAGAAGACGGCATACGAGATTTACGCACGTGACTGGAGTTCAGACGTGTGCTCTTCCGATCTCGACTCGGTGCCACTTTTTCAAG	GTGCGTAA

**Cr_1711**	CAAGCAGAAGACGGCATACGAGATTTGA<br>ATAGGTGACTGGAGTTCAGACGTGTGCTC<br>TTCCGATCTCGACTCGGTGCCACTTTTTC<br>AAG	CTATTCAA

Supplementary Table 8. Parameters for bioinformatics tools on Galaxy [12] used in the analysis of NGS reads (Cas12a)

Tool	Version	Parameters*
FastQC	v0.11.8	Default settings
Cutadapt	Galaxy Version 1.16.6 [13]	The 3 biological replicates of a given sample at a given time-point in the Cas12a screen always had the same reverse primer containing the Illumina barcode, and forward primers ILU1-F, ILU3-F and ILU4-F; or ILU2-F, ILU3-F and ILU4-F each containing different pseudo-barcodes. Thus Cutadapt was used to demultiplex biological replicates from each other.<br>▪ 5' (Front) anchored 6 bp pseudo-barcodes to be demultiplexed (-g): ^NNNNNN (refer to previous table for pseudo-barcode-forward primer association).<br>▪ Maximum error rate (--error-rate): 0.2<br>▪ Match times (--times): 1<br>▪ Minimum overlap length (--overlap): 4<br>▪ Multiple output: Yes (Each demultiplexed readset is written to a separate file)
Trimmomatic	v0.38	▪ HEADCROP: 29 (if amplified by ILU1-F); or 30 (if amplified by ILU2-F); or 32 (if amplified by ILU3-F); or 34 (if amplified by ILU4-F)<br>▪ CROP: 25
Bowtie2**	v2.4.2	▪ Number of allowed mismatches in seed alignment (-N): 1<br>▪ Length of the seed substring (-L): 21<br>▪ Function governing interval between seed substrings in multiseed alignment (-i): S,1,0.50<br>▪ Function governing maximum number of ambiguous characters (--n-ceil): L,0,0.15<br>▪ Alignment mode: end-to-end<br>▪ Number of attempts of consecutive seed extension events (-D): 20<br>▪ Number of times re-seeding occurs for repetitive reads: 3<br>▪ Save mapping statistics: Yes

* All parameters other than those mentioned here are kept at default values.
** Bowtie2 usage needs a genome fasta file for alignment. Nontargeting sgRNA and any other sgRNA that Bowtie2 could not find within the original CLIB89 genome file were appended as an extra chromosome so that Bowtie could align all sgRNA for the purposes of generating counts.

Supplementary Table 9. Parameters for bioinformatics tools on Galaxy [12] used in the analysis of NGS reads (Cas9)

**Tool**	**Version**	**Parameters***
FastQC	v0.11.8	Default settings
Cutadapt	Galaxy Version 1.16.6 [13]	Cutadapt was used to demultiplex samples containing the same Illumina barcode, but different pseudobarcodes at the 5’ end of the read. Samples were amplified with reverse primers Cr1669-1673;Cr1709-1711 and forward primers Cr1665-1668 each containing a different pseudo barcode as mentioned in Table<br>▪ 5’ (Front) anchored 6 bp pseudo-barcodes to be demultiplexed (-g): ^NNNNNN (refer to previous table for pseudo-barcode-forward primer association).<br>▪ Maximum error rate (--error-rate): 0.2<br>▪ Match times (--times): 1<br>▪ Minimum overlap length (--overlap): 4<br>▪ Multiple output: Yes (Each demultiplexed readset is written to a separate file)
Trimmomatic	v0.38	▪ HEADCROP: 30 (if amplified by Cr1665); or 32 (if amplified by Cr1666); or 34 (if amplified by Cr1667); or 36 (if amplified by Cr1668)<br>▪ CROP: 20
Bowtie2**	v2.4.2	▪ Number of allowed mismatches in seed alignment (-N): 1<br>▪ Length of the seed substring (-L): 19<br>▪ Function governing interval between seed substrings in multiseed alignment (-i): S,1,0.50<br>▪ Function governing maximum number of ambiguous characters (--n-ceil): L,0,0.15<br>▪ Alignment mode: end-to-end<br>▪ Number of attempts of consecutive seed extension events (-D): 20<br>▪ Number of times re-seeding occurs for repetitive reads: 3<br>▪ Save mapping statistics: Yes

* All parameters other than those mentioned here are kept at default values.
** Bowtie2 usage needs a genome fasta file for alignment. Nontargeting sgRNA and any other sgRNA that Bowtie2 could not find within the original CLIB89 genome file were appended as an extra chromosome so that Bowtie could align all sgRNA for the purposes of generating counts.

Supplementary Table 10. List of sgRNA (& associated cutting scores) used for validation of essential genes, non-essential genes, and significant genes for salt tolerance.

Gene	sgRNA ID (from Cas9 lib.)	sgRNA sequence	Cutting score (CS)
YALI1_B20188g	YALI1_B20188g_3	TTGCATCCTGATCGAAACCA	6.88
YALI1_D06665g	YALI1_D06665g_6	GGATGCTGCTACTTCCAAAT	5.73
YALI1_E17613g	YALI1_E17613g_6	CTTTGCACACCCCGTCAATT	7.14
YALI1_F08292g	YALI1_F08292g_3	GAACTCGTCAGCGAGCACGG	6.07
YALI1_F27686g	YALI1_F27686g_6	GCAGAAGAACCGCCTCACCA	6.90
YALI1_E23184g	YALI1_E23184g_3	CGAGTCGCCGACAACTGTAA	7.52
YALI1_A21345g	YALI1_A21345g_1	TCAATAGTAGCCTCAGACAA	6.80
YALI1_A03069g	YALI1_A03069g_5	TGCATCGGCGATATGTTCCA	6.10
YALI1_B00908g	YALI1_B00908g_5	GTTCTACGAGACCGATCACC	7.27
YALI1_C08600g	YALI1_C08600g_3	AATGGGGTCGAACGAAACGC	6.07
YALI1_D03952g	YALI1_D03952g_6	CTCCTGAGCGGCCTTCCACG	6.59
YALI1_D14276g	YALI1_D14276g_2	CTGGATCTCCAGCTGTACCG	6.35
YALI1_B03043g	YALI1_B03043g_2	AATGTCGCTCTGGTGAGTGA	6.64
YALI1_E18269g	YALI1_E18269g_3	ACACGCACTCAGTAAGGCAG	6.25
YALI1_F34105g	YALI1_F34105g_6	GAACGCCGTGATCATCGGAC	6.72
YALI1_E24201g	YALI1_E24201g_4	GACGTGGGCAAGAAAAAGGA	6.12
YALI1_C11819g	YALI1_C11819g_5	GTTTTGCCAGTTCCCCAACG	5.58
YALI1_A07277g	YALI1_A07277g_4	TGGCGGAGATCTAGATGTCG	7.11
YALI1_F10122g	YALI1_F10122g_1	CAGAAGGGAAAGTAGTACCG	5.90
YALI1_F09056g	YALI1_F09056g_5	CCGAGAAAACGGCCAAAGGG	5.86
YALI1_E23961g	YALI1_E23961g_2	AGGCTACTCGGGAGGAAACA	5.73
YALI1_F12478g	YALI1_F12478g_6	ACTTGTGCGCGCCTCCCACT	5.90

Supplementary References

1. Allen, F. *et al.* JACKS: joint analysis of CRISPR/Cas9 knockout screens. *Genome Res.* **29**, 464–471 (2019).
2. Li, W. *et al.* Quality control, modeling, and visualization of CRISPR screens with MAGeCK-VISPR. *Genome Biol.* **16**, 281 (2015).
3. Daley, T. P. *et al.* CRISPhieRmix: a hierarchical mixture model for CRISPR pooled screens. *Genome Biol.* **19**, 159 (2018).
4. Patterson, K. *et al.* Functional genomics for the oleaginous yeast Yarrowia lipolytica. *Metab. Eng.* **48**, 184–196 (2018).
5. Baisya, D., Ramesh, A., Schwartz, C., Lonardi, S. & Wheeldon, I. Genome-wide functional screens enable the prediction of high activity CRISPR-Cas9 and -Cas12a guides in Yarrowia lipolytica. *Nat. Commun.* **13**, 922 (2022).
6. Luo, J., Chen, W., Xue, L. & Tang, B. Prediction of activity and specificity of CRISPR-Cpf1 using convolutional deep learning neural networks. *BMC Bioinformatics* **20**, 332 (2019).
7. Ramesh, A., Ong, T., Garcia, J. A., Adams, J. & Wheeldon, I. Guide RNA Engineering Enables Dual Purpose CRISPR-Cpf1 for Simultaneous Gene Editing and Gene Regulation in. *ACS Synth. Biol.* **9**, 967–971 (2020).
8. Schwartz, C. M., Hussain, M. S., Blenner, M. & Wheeldon, I. Synthetic RNA Polymerase III Promoters Facilitate High-Efficiency CRISPR-Cas9-Mediated Genome Editing in Yarrowia lipolytica. *ACS Synth. Biol.* **5**, 356–359 (2016).
9. Schwartz, C., Shabbir-Hussain, M., Frogue, K., Blenner, M. & Wheeldon, I. Standardized Markerless Gene Integration for Pathway Engineering in Yarrowia lipolytica. *ACS Synth. Biol.* **6**, 402–409 (2017).

10. Schwartz, C. *et al.* Validating genome-wide CRISPR-Cas9 function improves screening in the oleaginous yeast Yarrowia lipolytica. *Metab. Eng.* **55**, 102–110 (2019).
11. Schwartz, C., Frogue, K., Ramesh, A., Misa, J. & Wheeldon, I. CRISPRi repression of nonhomologous end-joining for enhanced genome engineering via homologous recombination in Yarrowia lipolytica. *Biotechnol. Bioeng.* **114**, 2896–2906 (2017).
12. Galaxy Community. The Galaxy platform for accessible, reproducible and collaborative biomedical analyses: 2022 update. *Nucleic Acids Res.* (2022) doi:10.1093/nar/gkac247.
13. Jalili, V. *et al.* The Galaxy platform for accessible, reproducible and collaborative biomedical analyses: 2020 update. *Nucleic Acids Res.* **48**, W395–W402 (2020).
