# Supplementary material for: acCRISPR: an activity-correction method for improving the accuracy of CRISPR screens
Source: Commun Biol. 2023 Jun 8;6:617. doi: 10.1038/s42003-023-04996-8 (PMC10250353; doi:10.1038/s42003-023-04996-8)
Supplement: Supplementary file 3 — Description of Additional Supplementary Files [file 42003_2023_4996_MOESM3_ESM.pdf]

Description of Additional Supplementary Files

File name: Supplementary Data 1
Description: Raw sgRNA abundances from Cas9 screen.

File name: Supplementary Data 2
Description: Raw sgRNA abundances from Cas12a screen.

File name: Supplementary Data 3
Description: sgRNA cutting and fitness scores from Cas9 and Cas12a screens.

File name: Supplementary Data 4
Description: Essential and non-essential genes from Cas9 and Cas12a screens.

File name: Supplementary Data 5
Description: GO-enrichment test results for Cas9 essential genes.

File name: Supplementary Data 6
Description: GO-enrichment test results for Cas12a essential genes.

File name: Supplementary Data 7
Description: Y. lipolytica consensus set of essential genes.

File name: Supplementary Data 8
Description: GO-enrichment test results for consensus essential gene set.

File name: Supplementary Data 9
Description: sgRNA activity prediction scores for Cas9 library.

File name: Supplementary Data 10
Description: sgRNA activity prediction scores for Cas12a library.

File name: Supplementary Data 11
Description: Significant and non-significant gene hits from high salt tolerance screens.

File name: Supplementary Data 12
Description: GO-enrichment test results for essential genes unique to each of the 3 screens.

File name: Supplementary Data 13
Description: Replicate correlations for Cas9 and Cas12a screens.

File name: Supplementary Data 14
Description: NGS read demultiplexing information for Cas9 and Cas12a data.

File name: Supplementary Data 15
Description: Raw sgRNA abundances for untransformed Cas9 and Cas12a libraries.

File name: Supplementary Data 16
Description: Source data for figures.
